# Association of the *LEP* gene with immune infiltration as a diagnostic biomarker in preeclampsia

**DOI:** 10.3389/fmolb.2023.1209144

**Published:** 2023-08-10

**Authors:** Shaorong Chen, Yumin Ke, Weihong Chen, Sijia Wu, Xuanxuan Zhuang, Qiuya Lin, Qirong Shi, Zhuna Wu

**Affiliations:** Department of Gynecology and Obstetrics, The Second Affiliated Hospital of Fujian Medical University, Quanzhou, Fujian, China

**Keywords:** preeclampsia, *LEP* gene, immune infiltration, diagnostic marker, CIBERSORT

## Abstract

**Objective:** Preeclampsia (PE) is a serious condition in pregnant women and hence an important topic in obstetrics. The current research aimed to recognize the potential and significant immune-related diagnostic biomarkers for PE.

**Methods:** From the Gene Expression Omnibus (GEO) data sets, three public gene expression profiles (GSE24129, GSE54618, and GSE60438) from the placental samples of PE and normotensive pregnancy were downloaded. Differentially expressed genes (DEGs) were selected and determined among 73 PE and 85 normotensive control pregnancy samples. The DEGs were used for Gene Ontology (GO), Kyoto Encyclopedia of Genes and Genomes (KEGG), Disease Ontology (DO) enrichment analysis, and Gene Set Enrichment Analysis (GSEA). The candidate biomarkers were identified by the least absolute shrinkage and selection operator (LASSO) and support vector machine recursive feature elimination (SVM-RFE) analysis. The receiver operating characteristic curve (ROC) was applied to evaluate diagnostic ability. For further confirmation, the expression levels and diagnostic value of biomarkers in PE were verified in the GSE75010 data set (80 PE and 77 controls) and validated by qRT-RCR, Western blot, and immunohistochemistry (IHC). The CIBERSORT algorithm was used to calculate the compositional patterns of 22 types of immune cells in PE.

**Results:** In total, 15 DEGs were recognized. The GO and KEGG analyses revealed that the DEGs were enriched in the steroid metabolic process, receptor ligand activity, GnRH secretion, and neuroactive ligand−receptor interaction. The recognized DEGs were primarily implicated in cell-type benign neoplasm, kidney failure, infertility, and PE. Gene sets related to hormone activity, glycosylation, multicellular organism process, and response to BMP were activated in PE. The *LEP* gene was distinguished as a diagnostic biomarker of PE (AUC = 0.712) and further certified in the GSE75010 data set (AUC = 0.850). The high expression of *LEP* was associated with PE in clinical samples. In addition, the analysis of the immune microenvironment showed that gamma delta T cells, memory B cells, M0 macrophages, and regulatory T cells were positively correlated with *LEP* expression (*P* < 0.05).

**Conclusion:** *LEP* expression can be considered to be a diagnostic biomarker of PE and can offer a novel perspective for future studies regarding the occurrence and molecular mechanisms of PE.

## Introduction

Immune system dysregulation can influence maternal, postnatal, or fetal immune progression. Preeclampsia (PE) is a common and unique pregnancy-associated multisystem disorder with an immune basis in humans and features placental malperfusion (Hu et al., 2019). PE is defined as the onset of hypertension (>140/90 mmHg) and proteinuria (>0.3 g/24 h) after 20 weeks of gestation (Armaly et al., 2018), which affects 3%–5% of pregnancies worldwide and is a leading cause of maternal mortality (Mol et al., 2016). An estimated 15% of preterm births worldwide are attributable to PE (Mol et al., 2016). Because effective treatments are lacking, the identification of novel early diagnostic biomarkers and therapeutic targets is crucial for improving adverse outcomes for the mother and fetus in PE (Maric-Bilkan et al., 2019).

In the past few years, integrated microarray technology with the bioinformatics analysis method could identify novel genes that might serve as diagnostic and prognostic biomarkers in various diseases (Wu et al., 2019; Zhao Y. et al., 2020; Sheng et al., 2020; Zhao et al., 2021). For instance, four immune-related genes (*CRH*, *PI3*, *CCL18*, and *CCL2*) were selected from the random forest model to construct a nomogram to predict PE (Wang et al., 2020). Compared to preterm controls, patients with early onset PE showed downregulated expression levels of the placental mesoderm-specific transcript (*MEST*) and necdin (*NDN*) genes (Deyssenroth et al., 2020). Furthermore, an increasing number of studies have reported the crucial role of immune cell infiltration in the occurrence and development of various diseases (Zhao Y. et al., 2020; Ballot et al., 2020; Deng et al., 2020; Deng et al., 2021). PE basically develops in two stages: an abnormal maternal immune system response in early pregnancy and a later maternal systemic inflammatory response (Roberts and Hubel, 2009; Redman and Staff, 2015). The ratio of circulating Th1 (T-helper type-1 lymphocytes)/Th2 (T-helper type-2 lymphocyte response) in PE is higher than it is in normal pregnant subjects in their third trimester (Saito et al., 1999). Uterine natural killer cells and macrophages have been revealed to activate multiorgan endothelial cells, followed by clinical symptoms of PE (Nagamatsu and Schust, 2010; Acar et al., 2011). To our knowledge, no study has yet employed CIBERSORT to delve into infiltrating immune cells in PE and further identify early diagnostic biomarkers for PE.

Therefore, the goal of this study was to discover the novel diagnostic immune-related genes related to PE to identify diagnostic markers for PE, based on machine-learning algorithms and the logistic regression method. In this study, to our knowledge, CIBERSORT has been applied for the first time to compute the quotas of infiltrating immune cells among PE and normotensive pregnancy (control) placental samples. Finally, the association among recognized diagnostic markers and infiltrating immune cells was explored to facilitate further research in this area.

## Materials and methods

### Downloading and processing of microarray data

First, we obtained the matrix files of GSE24129, GSE54618, GSE60438, and GSE75010 from the Gene Expression Omnibus (GEO) database (https://www.ncbi.nlm.nih.gov/gds) (Table 1). On the basis of their probe annotation files, the probes were mapped to gene symbols in each data set. For multiple probes corresponding to the same gene, the average expression value of the gene was calculated to represent the gene expression level. After merging the three data sets into a metadata cohort and removing the batch effect, the “SVA” package of R software was applied (Leek et al., 2012). The background correction and normalization of raw data were processed by the limma package of R (http://www.bioconductor.org/), where genes with |log fold change (FC)|>0.5 and adjusted *p <* 0.05 were defined as DEGs*.* GSE75010 was used to determine and validate functions of significant DEGs (Table 1).

**TABLE 1:** GEO database data of the preeclampsia mRNA expression profile.

Data set ID	Platform	Preeclampsia	Normotensive control
Train group			
GSE24129	GPL6244-17930	8	8
GSE54618	GPL10558-50081	5	12
GSE60438	GPL6884; GPL10558	60	65
Test group			
GSE75010	GPL6244-17930	80	77

### Annotations of DEGs’ function and pathway

A total of 15 differentially expressed genes (DEGs) were analyzed using the R language with the clusterProfiler, org.Hs.eg.db, enrichplot, and ggplot2 packages via Gene Ontology (GO) and Kyoto Encyclopedia of Genes and Genomes (KEGG) analyses. *p* < 0.05 was considered to denote significant enrichment. The R package “clusterProfiler” and “DOSE” were applied to conduct the Disease Ontology (DO) enrichment analyses on DEGs. The Gene Set Enrichment Analysis (GSEA) was used to analyze the association between the risk score and hallmarks. The GSEA was applied to recognize the most important feature among the PE and normotensive pregnancy groups. The “c5.go.v7.4.symbols.gmt” was applied as the reference gene set from the Molecular Signatures Database (MSigDB). The gene set was considered to be significant at a *p* < 0.05.

### Screening candidate biomarker for diagnosis

We predicted disease status via two machine-learning algorithms. To increase the prediction accuracy, the least absolute shrinkage and selection operator (LASSO)*—*a regression-based analysis method that scrutinizes variable selection and regularization in PE models—was used. The R package “glmnet” was applied to conduct LASSO regression analysis on identification of valuable DEGs related to the discrimination of PE and normotensive control. The support vector machine (SVM) is an efficient and widely applied supervised machine-learning algorithm for disease classification and regression tasks (CORTES and Vapnik, 1995). Hence, conjugated LASSO and SVM-RFE were used to screen the overlapping genes, which were further verified in the GSE75010 data set.

### Predictive value of diagnostic markers in PE

The mRNA expression data were obtained from 73 PE and 85 normotensive pregnancy samples from the GEO database, which were used to generate the receiver operating characteristic (ROC) curves to determine the predictive value of biomarkers. The area under the ROC curve (AUC) was used to ascertain the diagnostic capability of biomarkers in distinguishing PE earlier from normotensive samples and was also verified by the GSE75010 data set.

### Construction of PPI network

To build a PPI network, we employed “LEPTIN” in the “protein name” module and “*Homo sapiens*” in the organism module to search from the STRING website (https://string-db.org/). We set the key parameters as follows: meaning of network edges (“confidence”), the minimum required interaction score [“highest confidence (0.900)”], and the maximum number of interactors to show (“no more than 10 interactors”) in the first shell. The STRING tool was used to perform KEGG and GO molecular function analyses of LEP-related genes.

### Evaluation of infiltrating immune level

From the gene expression profiles in PE, the CIBERSORT (http://cibersort.stanford.edu/) algorithm was used to quantify the proportion of immune infiltration cells. We downloaded a gene signature matrix with interpretation, known as the 22 kinds of immune cells (LM22) with 1,000 permutations, from the webpage of CIBERSORT to estimate the putative abundance of immune cells (Newman et al., 2015). The R package “corrplot” was applied to conduct the correlation and visualization of LM22. The R package “vioplot” was used to visualize the differences in immune cells between the PE and normotensive control groups. Pearson’s correlation analysis was used to explore the screened diagnostic biomarker relation to the levels of immune infiltration cells. The chart technique with R package “ggplot2” was applied to visualize the aforementioned result.

### Patient and tissue samples

A total of 32 paraffin-embedded PE and 41 normotensive specimens were diagnosed at the Second Affiliated Hospital of Fujian Medical University (Fujian, China) from August 2017 to September 2021. The research was approved by the Research Ethics Committee of the Second Affiliated Hospital of Fujian Medical University prior to the study.

### Immunohistochemistry

IHC staining was performed as previously described (Chen et al., 2016). The primary antibody was anti-leptin (Servicebio, Wuhan). Leptin staining intensity ratios were scored as follows: negative = 0, light yellow = 1, brownish yellow = 2, or tan = 3. The staining cells were scored as follows: less than 1/3 = 1, between 1/3 and 2/3 = 2, or more than 2/3 = 3. The final score for leptin expression was calculated by multiplying the two scores. Slides were divided into low- and high-expression groups, corresponding to scores of <3 or ≥3, respectively. The histopathological diagnosis of the patients was established by two pathologists specialized in obstetrics and gynecology.

### Quantitative real-time PCR

Total RNA was extracted from placental tissue immediately after normal labor and cesarean section utilizing TRIzol (TaKaRa, Japan), and then, cDNA was prepared according to the protocol (TaKaRa, Japan). The detailed procedure is presented in the Supplementary Methods. GAPDH was used as an internal reference and the relative mRNA expression of leptin was calculated by the 2^−ΔΔCT^ method. qRT-PCR for each sample was repeated in three independent experiments. The primer sequences are shown below:

GAPDH

Forward: 5′-CAT​GTT​CGT​CAT​GGG​TGT​GAA​CCA-3′,
Reverse: 5′-AGT​GAT​GGC​ATG​GAC​TGT​GGT​CAT-3′.

LEPTIN

Forward: 5′-AAC​GTG​ATC​CAA​ATA​TCC​AAC​G-3′,
Reverse: 5′-AGC​TCT​TAG​AGA​AGG​CCA​GCA-3’.

### Western blotting

Total protein was extracted from each 50 mg placenta sample using RIPA lysis buffer (CW Biotechnology, Beijing China) with protease (Solarbio, China), DNA enzyme inhibitor (Solarbio, China), and phosphatase inhibitors (CW Biotechnology, Beijing China). Western blotting was performed as described previously (Guo et al., 2016). The following antibodies were utilized: anti-leptin (Servicebio, China) and anti-GAPDH (Cell Signaling Technology, United States). The secondary antibody was anti-rabbit IgG (Cell Signaling Technology, United States). Gray values were measured with ImageJ.

### Statistical analysis

We used the R software (v.4.1.1) for all statistical analyses. The Mann–Whitney U test was used to compare the PE and normotensive control groups. The LASSO regression analysis, SVM algorithm, ROC curve analysis, Pearson’s correlation, and unpaired *t*-test were used as described above. *p* < 0.05 was considered to indicate statistically significant differences for all analyses.

## Results

### Study procedure

**FIGURE 1:**
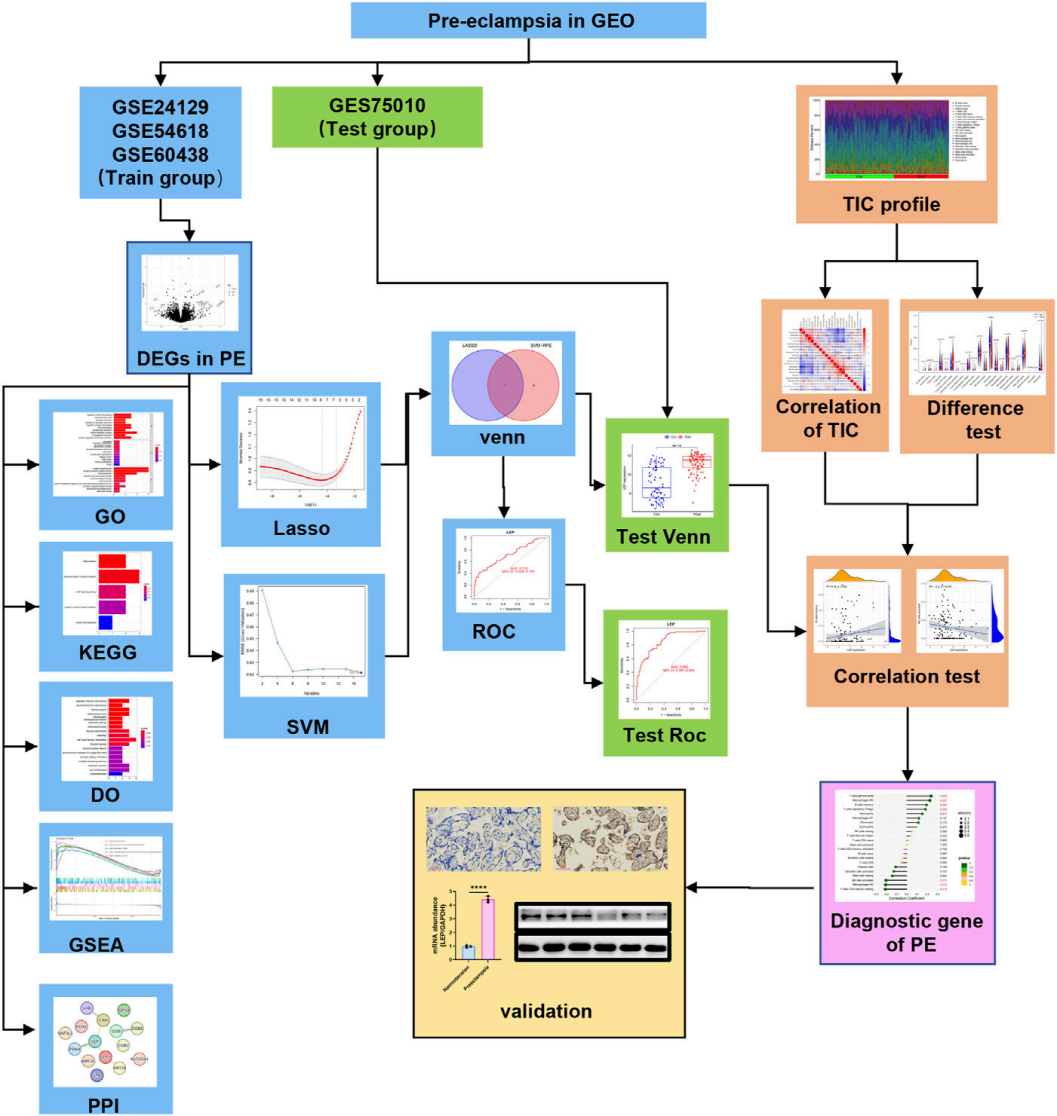
Analysis workflow of this study.

The analysis procedure used in the study is shown in Figure 1. We downloaded the transcriptome RNA-seq data from the GEO database. We identified DEGs between PE and normotensive control groups. We conducted GO, KEGG, DO, and GSEA analyses on DEGs. Conjugated LASSO and SVM-RFE were used to screen the overlapping candidate genes; the ROC curve was applied to determine the predictive value of the biomarkers, which were further validated in the GSE75010 data set. The CIBERSORT algorithm was used to calculate the compositional patterns of LM22 in PE. Correlation analysis was performed among the diagnostic markers and infiltrating immune cells.

### Identification of DEGs in PE

**FIGURE 2:**
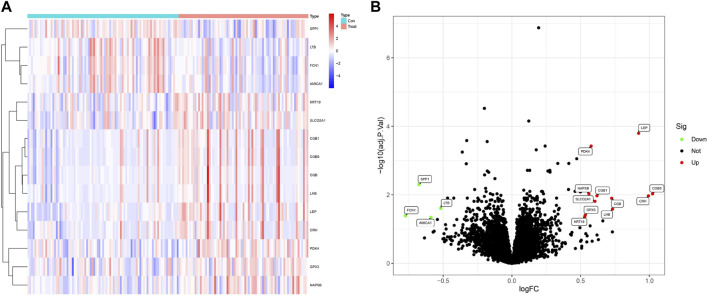
Identification of DEGs. **(A)** Heatmap plots of 15 DEGs between preeclampsia tissue and normotensive control pregnancy samples from the GEO database. Row name of heatmap is the gene name, and column name is the ID of samples which is not shown in the plot. Red to blue represent the expression level from high to low in the heatmaps. **(B)** Volcano plots of 15 DEGs between preeclampsia tissue and normotensive control pregnancy samples. Red dots in the volcano plots represent upregulation, green dots represent downregulation, and black dots represent genes without differential expression.

This study involved three data sets from the GEO database (GSE24129, GSE54618, and GSE60438) and included a total of 73 PE and 85 normotensive control pregnancy samples. We obtained a total of 15 DEGs when comparing PE and normotensive controls (Figures 2A, B). Of these genes, four were remarkably downregulated and 11 were markedly upregulated (Figure 2B).

### Functional enrichment and correlation analysis

**FIGURE 3:**
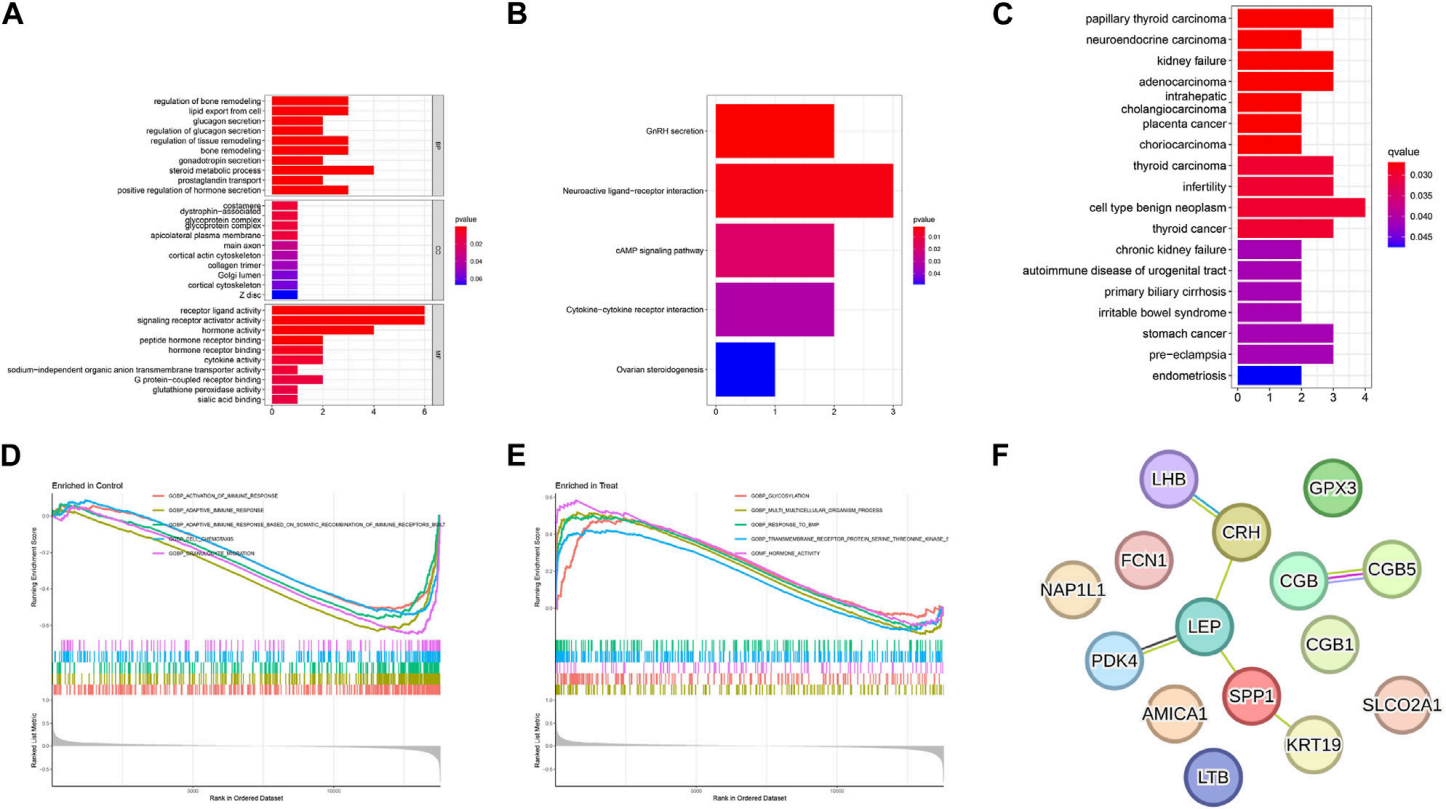
Functional enrichment analyses to identify potential biological processes. **(A)** GO analysis. GO analysis divided DEGs into three functional groups: molecular function (MF), biological processes (BP), and cell composition (CC). **(B)** KEGG analysis of DEGs. **(C)** Disease Ontology enrichment analysis of DEGs between preeclampsia and normotensive control pregnancy samples. **(D, E)** Enrichment analyses between preeclampsia and normotensive control pregnancy samples via Gene Set Enrichment Analysis. **(F)** Using the STRING tool to construct PPI networks exploring 15 DEGs binding protein interactions.

The results from the GO analysis showed that the DEGs were closely related to hormone-related GO terms, such as hormone activity, steroid metabolic process, and positive regulation of hormone secretion, and to receptor ligand–related GO terms, particularly receptor ligand activity, signaling receptor activator activity, and peptide hormone receptor binding (*P* < 0.05, Figure 3A). In addition, KEGG analysis showed enrichment of the GnRH secretion, neuroactive ligand–receptor interaction, cAMP signaling pathway, and cytokine–cytokine receptor interaction (*P* < 0.05, Figure 3B). The analysis of DO enrichment showed that DEGs were mostly related to cell-type benign neoplasm, infertility, PE, and kidney failure (Figure 3C). The GSEA results revealed that the enriched functions mainly enrolled activation of immune response, adaptive immune response, and cell chemotaxis in control (Figure 3D, Supplementary Table S1); and glycosylation, multicellular organism process, and hormone activity in PE (Figure 3E, Supplementary Table S2). These results conclusively show that immune response and hormone secretion have vital roles in PE. A PPI network with 15 DEGs was obtained. The STRING tool identified five binding proteins. *LEP*, *LHB*, *PDK4*, *SP1*, and *KRT19* are shown in Figure 3F.

### Recognition and validation of diagnostic biomarkers

**FIGURE 4:**
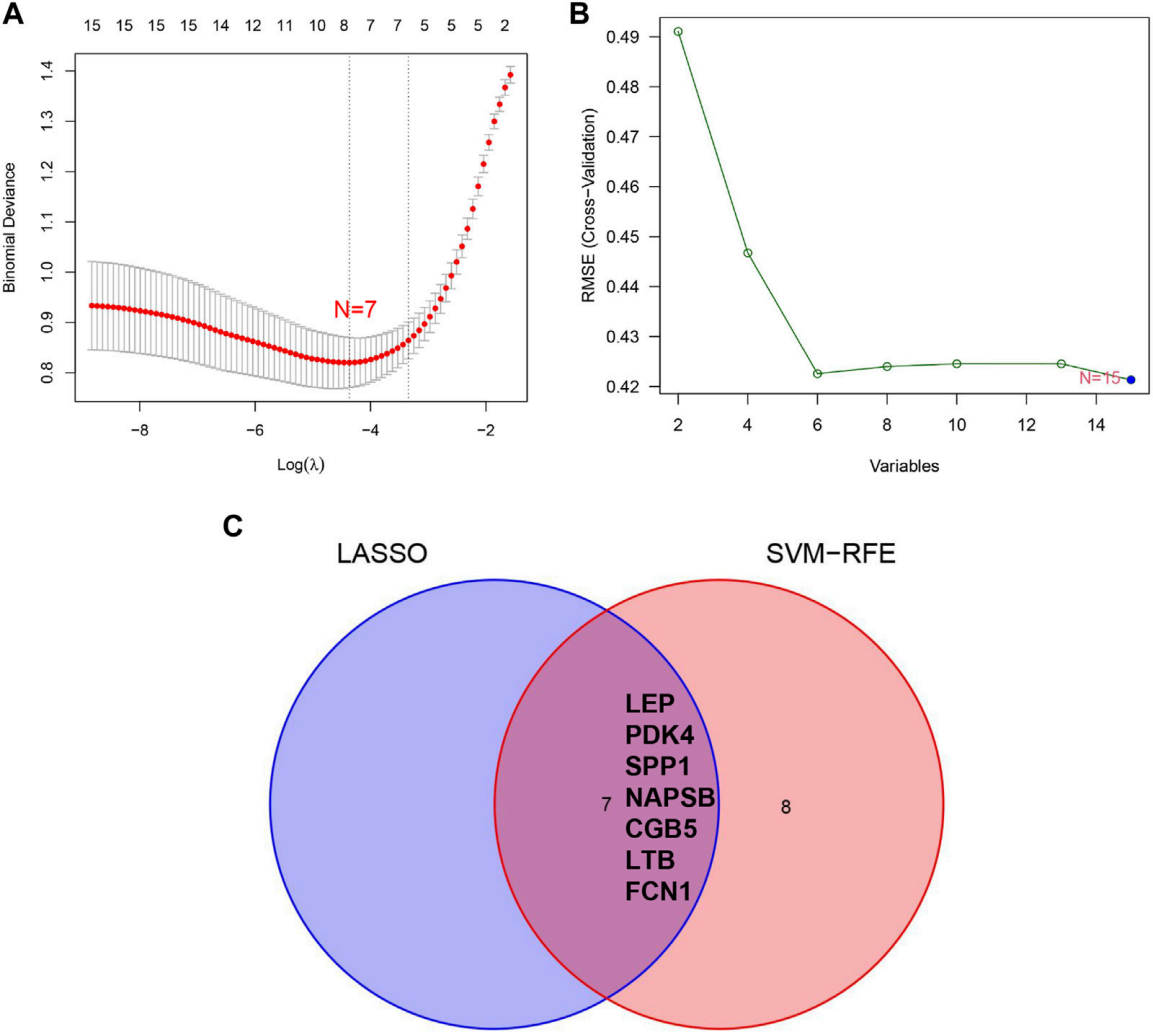
Screening process of diagnostic biomarker candidates for preeclampsia diagnosis. **(A)** Tuning feature selection in the least absolute shrinkage and selection operator model. **(B)** Plot of biomarker selection via support vector machine recursive feature elimination (SVM-RFE) algorithm. **(C)** Venn diagram demonstrating seven diagnostic markers shared by the least absolute shrinkage and selection operator and SVM-RFE algorithms.

**FIGURE 5:**
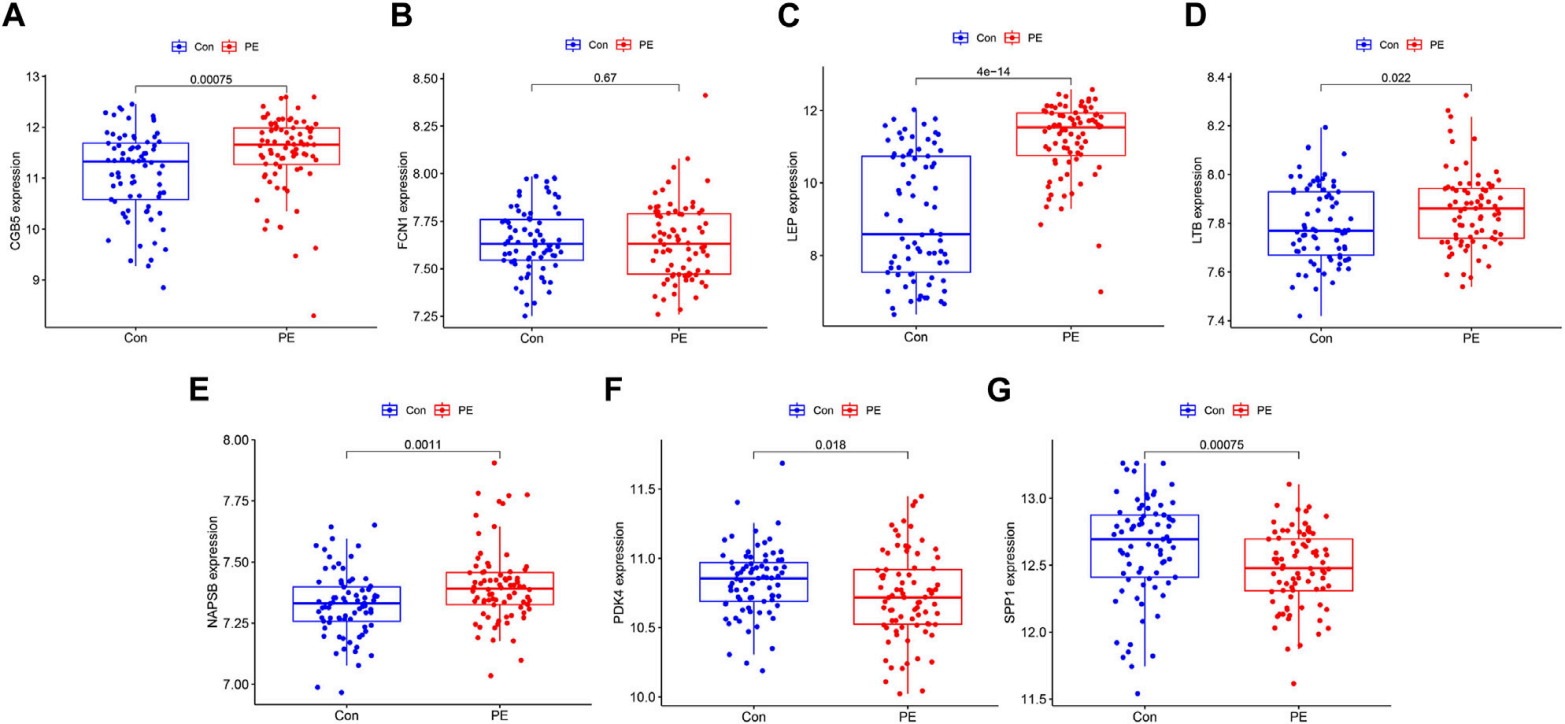
Validation of the expression of diagnostic biomarkers in the GSE75010 data set. **(A)** *CGB5*, **(B)** *FCN1*, **(C)** *LEP*, **(D)** *LTB*, **(E)** *NAPSB*, **(F)** *PDK4*, and **(G)** *SPP1*.

We applied the LASSO and SVM-RFE algorithm to selective potential biomarkers. The seven DEGs were identified as diagnostic biomarkers using LASSO regression for PE (Figure 4A). The five DEGs were verified by applying the SVM-RFE (Figure 4B). Between the two algorithms, the seven overlapping candidate genes (*LEP*, *PDK4*, *SPP1*, *NAPSB*, *CGB5*, *LTB*, and *FCN1*) were ultimately screened (Figure 4C). Additionally, to produce more reliable and accurate DEGs, verification of the seven DEGs’ expression levels was conducted using the GSE75010 data set. The CGB5, LEP, LTB, and NAPSB expression levels in PE samples were remarkably higher than in that of the normotensive group (Figures 5A, C–E; *p* < 0.05). The PDK4 and SPP1 expression levels in PE tissue were significantly lower than those in the normotensive group (Figures 5F, G; *p* < 0.05). However, the *FCN1* expression showed no significant difference between the two groups (Figure 5B). Hence, we next explored the potential value of the diagnostic model combination of the six identified DEGs by applying a logistic regression algorithm.

### Effectiveness of featured diagnostic biomarkers in PE

**FIGURE 6:**
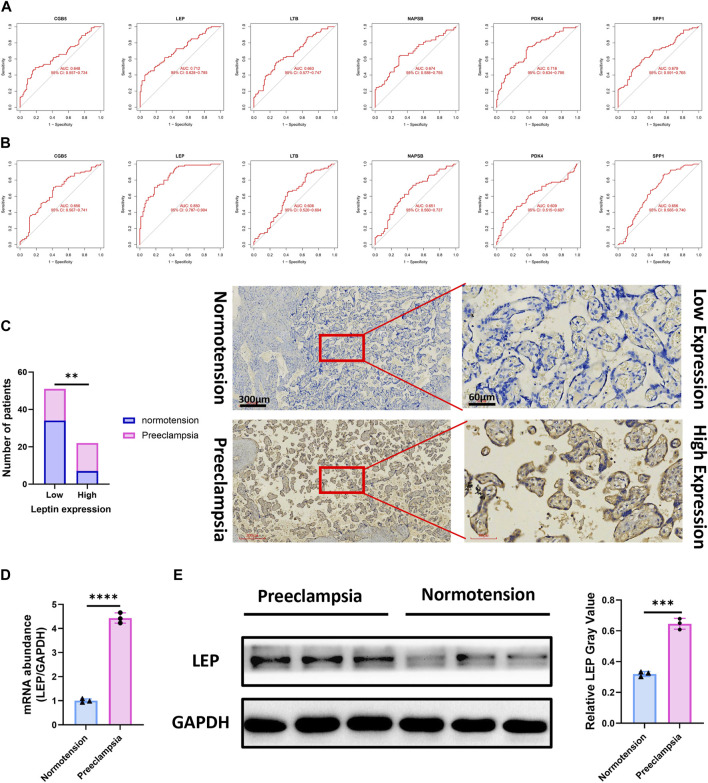
Receiver operating characteristic (ROC) curve of the diagnostic effectiveness of the six diagnostic markers. **(A)** ROC curve of *CGB5*, *LEP*, *LTB*, *NAPSB*, *PDK4*, and *SPP1* after fitting to one variable in the metadata cohort; **(B)** ROC curve of *CGB5*, *LEP*, *LTB*, *NAPSB*, *PDK4*, and *SPP1* after fitting to one variable in the GSE75010 data set. **(C)** Significantly high LEP expression was observed in PE tissues when compared with normotension specimens (PE = 32; normotension = 41). Representative images (×40 and ×200) of IHC staining for LEP in 32 PE and 41 normotensive patients (high expression vs. low expression). **(D)** qRT-PCR of *LEP* expression in PE placental tissue when compared with normotension specimens. **(E)** WB analysis of *LEP* expression in PE placental tissue when compared with normotension specimens. Scale bars are shown. *p < 0.05. *p*-values were calculated by chi-square tests.

Both *LEP* (AUC = 0.712) and *PDK4* (AUC = 0.718) showed a good diagnostic value for the early diagnosis of PE (Figure 6A). Furthermore, a forceful discerning capacity was verified in the GSE75010 data set with an AUC of 0.850 in *LEP* (Figure 6B), showing that the *LEP* gene had a higher diagnostic capacity. We assessed the expression of *LEP* across PE and normotensive tissues via immunohistochemistry and found that high expression of *LEP* was associated with PE (Figure 6C; *p* < 0.05). For further clinical validation, we assessed the expression of *LEP* using qRT-PCR and revealed that high expression of *LEP* was associated with PE (Figure 6D; *p* < 0.05). We performed Western blotting to assess that *LEP* is upregulated in PE and found that high expression of *LEP* was associated with PE (Figure 6E; *p* < 0.05). The aforementioned results indicate that the *LEP* gene had a higher diagnostic capacity.

### Building PPI network

**FIGURE 7:**
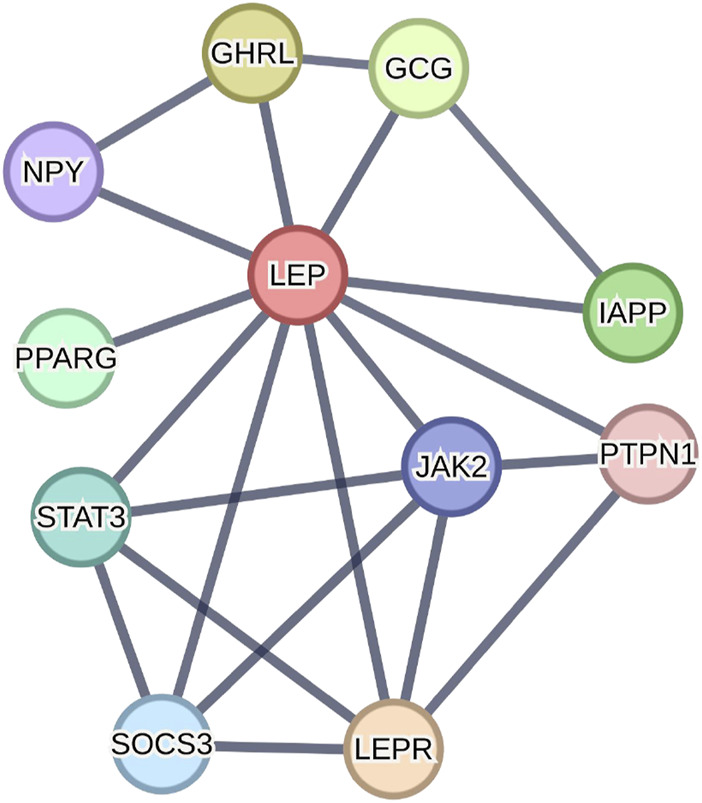
Using the STRING tool to construct PPI networks exploring leptin-binding protein interactions.

A PPI network with 14 nodes was obtained. The STRING database analysis identified 10 highest leptin-binding proteins. As shown in Figure 7, *GCG*, *IAPP*, *LEPR*, *GHRL*, *STAT3*, *PPARG*, *SOCS3*, *NPY*, *JAK2*, and *PTPN1* were predicted to have the most powerful interactions with leptin. And then we performed the GO molecular function and KEGG analyses of the proteins predicted via the STRING tool. The GO_MF analysis showed significant differences in the relevance of the signaling receptor binding and hormone activity (Table 2). KEGG analysis showed significant differences in association with the adipocytokine and JAK-STAT signaling pathways (Table 3). The KEGG analysis revealed that *STAT3*, *SOCS3*, *LEPR*, *JAK2*, and *NPY* were associated with leptin in the adipocytokine signaling pathway, and *STAT3*, *SOCS3*, *LEPR*, and *JAK2* were associated with leptin in the JAK-STAT signaling pathway, suggesting that leptin may interact with these proteins to activate both adipocytokine and JAK/STAT signaling in PE.

**TABLE 2:** Leptin GO molecular function (STRING tools).

Term ID	Term description	Genes	*p*-value
GO:0005102	Signaling receptor binding	9	1.66E-05
GO:0005179	Hormone activity	5	1.66E-05
GO:0001664	G protein–coupled receptor binding	5	0.00037
GO:0051427	Hormone receptor binding	4	0.0023
GO:0005515	Protein binding	11	0.0052
GO:0019903	Protein phosphatase binding	3	0.026
GO:0051428	Peptide hormone receptor binding	2	0.026
GO:0098772	Molecular function regulator	8	0.026
GO:0001103	RNA polymerase II repressing transcription factor binding	2	0.0471

**TABLE 3:** Leptin KEGG pathway (STRING tools).

Term ID	Term description	Genes	*p*-value
hsa04920	Adipocytokine signaling pathway	6	3.94E-10
hsa04080	Neuroactive ligand–receptor interaction	6	1.77E-06
hsa04630	JAK-STAT signaling pathway	5	1.99E-06
hsa04935	Growth hormone synthesis, secretion, and action	4	3.85E-05
hsa04917	Prolactin signaling pathway	3	0.00052
hsa04931	Insulin resistance	3	0.0015
hsa04152	AMPK signaling pathway	3	0.0018
hsa04932	Non-alcoholic fatty liver disease	3	0.003
hsa04024	cAMP signaling pathway	3	0.0071
hsa01521	EGFR tyrosine kinase inhibitor resistance	2	0.0298
hsa05235	PD-L1 expression and PD-1 checkpoint pathway in cancer	2	0.0342
hsa04659	Th17 cell differentiation	2	0.0386
hsa04933	AGE-RAGE signaling pathway in diabetic complications	2	0.0386
hsa05145	Toxoplasmosis	2	0.0386
hsa04380	Osteoclast differentiation	2	0.0472

### *LEP* is associated with percentage of immune cells

**FIGURE 8:**
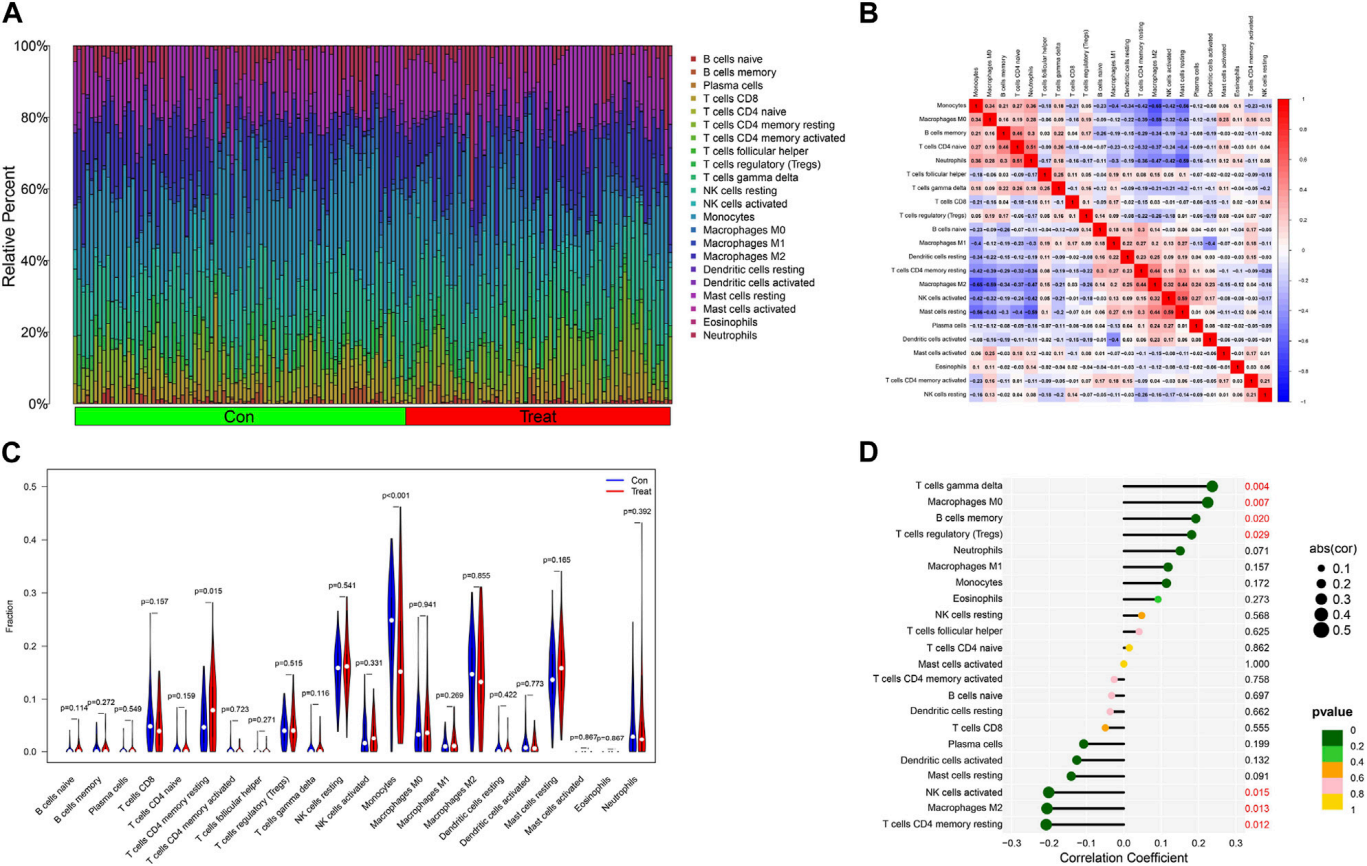
Distribution and visualization of immune cell infiltration and correlation analysis. **(A)** Barplot showing the proportion of 22 immune cell subtypes between preeclampsia tissue and normotensive control pregnancy samples. **(B)** Heatmap showing the correlation matrix of all 22 immune cell subtype compositions. Both horizontal and vertical axes demonstrate immune cell subtypes. Immune cell subtype compositions (higher, lower, and same correlation levels are displayed in red, blue, and white, respectively), and Pearson coefficient was used for the significance test. **(C)** Violin plot shows the ratio differentiation of 22 kinds of immune cells between preeclampsia tissue and normotensive control pregnancy samples, and Wilcoxon rank sum was used for the significance test. **(D)** Correlation between LEP and infiltrating immune cells in preeclampsia.

To further verify the relationship between the *LEP* gene and immune cell infiltration, we first applied the CIBERSORT algorithm to determine the proportions of the 22 types of infiltrating immune cells in the PE and control samples (Figures 8A, B; Supplementary Table S3). Next, the component of immune cells in PE vs. normotensive group was explored. The ratio of monocytes in PE was significantly lower than that in the normotensive control (*P* < 0.001). However, the ratio of CD4^+^ resting memory T cells (*p* = 0.015) in PE was significantly higher than that in normotensive controls (Figure 8C). Furthermore, we studied the relationship between the *LEP* gene and infiltrating immune cells. *LEP* was positively correlated with gamma delta T cells (*r* = 0.237, *p* < 0.05), M0 macrophages (*r* = 0.224, *p* < 0.05), memory B cells (*r* = 0.192, *p* < 0.05), and regulatory T cells (*r* = 0.181, *p* < 0.05), while being negatively correlated with resting CD4 memory T cells (*r* = −0.208, *p* < 0.05), M0 macrophages (*r* = −0.205, *p* < 0.05), and activated NK cells (*r* = −0.200, *p* < 0.05) (Figure 8D). The impact of the *LEP* gene was supported by these results regarding immune activity.

## Discussion

PE is a hypertensive disorder specific to pregnancy and is one of the main causes of maternal and fetal mortality and morbidity worldwide*.* PE is accompanied by fetal growth restriction, preterm birth, and other severe complications that result in large economic and mental health costs to the affected households’ families and society. It is well known that maternal immune tolerance plays a key role in the pathogenesis of PE, and the infiltration of placental immune cells is closely associated with spiral artery remodeling. Poor remodeling of the spiral arteries can induce placental ischemia and hypoxia, further contributing to the development and progression of PE (Zarate et al., 2014). A growing number of studies have investigated the role of infiltrating immune cells as a new bioinformatic method to scrutinize the diagnosis and prognosis of various diseases such as breast cancer (Yao et al., 2021), gastric cancer (Xiang et al., 2020), and osteosarcoma (Chi Zhang et al., 2020), as well as acute myocardial infarction (Zhao E. et al., 2020). Consequently, several studies have vigorously sought novel molecular biomarkers and explored immune cell infiltration in PE, which could improve the poor outcomes of PE patients in the clinic. Recently, circular RNAs and mRNA have been investigated as potential biomarkers in pregnancy, especially with respect to their molecular mechanisms (Triche et al., 2014; Huang et al., 2018; Kang et al., 2021), therapeutic targets (Deng et al., 2019), and subclassification (Leavey et al., 2015) in PE. However, there are very few studies on the immune cell infiltration association with DEGs in PE. Hence, we focused on the identification of significant diagnostic DEGs for PE and identified the role of infiltrating immune cells in PE.

To our knowledge, this is the first study to fully utilize multiple GEO data sets for knowledge mining by a machine*-*learning approach in PE and remarkedly identify diagnostic biomarkers related to infiltrating immune cells. In all, 15 DEGs were identified between the PE and normotensive control groups. The analysis of the 15 DEGs’ disease enrichment was mostly related to cell-type benign neoplasm, infertility, PE, and kidney failure. The results of the GSEA showed that the enriched GO terms generally involved immune response and hormone activity such as the glycosylation and transmembrane receptor protein serine threonine kinase signaling pathway. A successful pregnancy mandatorily requires well-balanced hormone levels and appropriate immune responses*.* These results are in line with our findings, generally involving glycosylation and hormone and immune responses, collectively and mutually participating in the diagnosis and pathogenesis of PE. The trophoblasts and endothelial cells can produce asymmetrically glycosylated IgG and react with the Fc portion of human immunoglobulin molecules and certain leukocytes in the cytoplasm and cell membrane. The study indicated that the Fab fragment of glycosylated IgG may act as the key component in placental immune evasion (Gu et al., 2015). PE is characterized by lower levels of serum hyperglycosylated hCG (hCG-H) at 8–13 weeks’ gestation (Keikkala et al., 2013). However, in the second trimester of pregnancy, low urine hCG-H appeared to predict subsequent PE (Bahado-Singh et al., 2002) but not in the serum samples (Keikkala et al., 2014). Hence, bioinformatics methods were applied to identify the novel diagnostic biomarkers of PE related to immune cell infiltration that may contribute to its early diagnosis and further therapy.

We identified a diagnostic biomarker based on combining two machine-learning algorithms and further using diagnostic ability analysis, and further verified these in the GSE75010 data set. Leptin protein is encoded by the *LEP* gene in humans, which is located on chromosome 7q31. This hormone is significantly associated with adiposity in humans (Havel et al., 1996). Additionally, leptin may play a part in the female reproductive system (DarrellBrann et al., 2002). Leptin participates in the interplay of metabolism, inflammation, and immune system disorders (Abella et al., 2017). *LEP* gene mutation decreases leptin concentration and increases type 2 diabetes mellitus and obesity (Qadir and Ahmed, 2017). Higher circulating leptin and the AA genotype of −2548 G/A polymorphism of the *LEP* gene may be associated with PE/pregnancy-induced hypertension (Sugathadasa et al., 2010). *LEP* gene hypomethylation in the placenta and elevated leptin concentration in maternal blood were observed in early onset PE (Hogg et al., 2013; Xiang et al., 2013). Hence, based on these results, we believe that *LEP* may play an important role in the occurrence and development of PE.

We applied the CIBERSORT method to evaluate the types of infiltrating immune cells in PE and normotensive controls. We detected a decreased infiltration of monocytes, and increased infiltration of CD4^+^ resting memory T cells was potentially correlated with the occurrence and development of PE. Additionally, we found that the *LEP* gene was related to gamma delta T cells, M0 macrophages, memory B cells, regulatory T cells, CD4 memory resting T cells, M0 macrophages, and activated NK cells. The first pathogenic step of PE is an abnormal immune response to the placenta against the maternal immune system; this is followed by the subsequent appearance of a systemic inflammatory response enrolling the endothelium (Veenstra van Nieuwenhoven et al., 2003). Thus far, previous studies have mainly focused on some immune cells such as T cells (Salmon et al., 2011), natural killer (NK) cells (Borzychowski et al., 2005; Acar et al., 2011), and macrophages (Hayashi et al., 2004; Nagamatsu and Schust, 2010) in PE. Furthermore, low-dose aspirin (LDA) is the most researched and utilized drug to prevent PE. Aspirin has anti-inflammatory, anti-oxidant, and immuno-modulatory functions (Parkinson, 2006; Nascimento-Silva et al., 2007). Our findings together with the evidence mentioned earlier have shown that the *LEP* gene associated with several types of immune cell infiltration plays an important role in PE and should be the focus of future experimental work.

Our study has some limitations. First, important and integrated clinical material was not obtained from the retrospective study, such as complications, birth outcomes, and past medical history. Second, the number of cases in the GSE24129 and GSE54618 data sets was low. Additionally, the reproducibility and function of the *LEP* gene and related immune cell infiltration in PE should be further validated by prospective studies with larger sample sizes.

## Conclusion

Based on the GEO database, we analyzed the immune characteristics of PE and identified that the *LEP* gene acts as a novel diagnostic biomarker of PE. The abnormal expression of immune-related genes may change the immune response by facilitating the infiltration of immune cells, which could influence the early identification, occurrence, and development of PE.

## Data Availability

The original contributions presented in the study are included in the article/Supplementary Material; further inquiries can be directed to the corresponding authors.
