# Supplementary material for: Association of the LEP gene with immune infiltration as a diagnostic biomarker in preeclampsia
Source: Front Mol Biosci. 2023 Aug 10;10:1209144. doi: 10.3389/fmolb.2023.1209144 (PMC10448764; doi:10.3389/fmolb.2023.1209144)
Supplement: Supplementary file 1 [file Table1.docx]

**Supplementary Table S1: Enrichment analyses via gene set enrichment analysis in control group**

| Enrichment Description | pvalue | p.adjust |
| --- | --- | --- |
| GOBP_ activation of immune response | 1.00E-10 | 2.35E-08 |
| GOBP_ adaptive immune response | 1.00E-10 | 2.35E-08 |
| GOBP_ adaptive immune response based on somatic recombination of receptor built from immunoglobulin superfamily domains | 1.00E-10 | 2.35E-08 |
| GOBP_ cell chemotaxis | 1.00E-10 | 2.35E-08 |
| GOBP_ granulocyte migration | 1.00E-10 | 2.35E-08 |

**Supplementary Table S2: Enrichment analyses via gene set enrichment analysis in PE group**

| Enrichment Description | pvalue | p.adjust |
| --- | --- | --- |
| GOBP_ glyocosylation | 1.75E-06 | 0.0001 |
| GOBP_ multi multicellular organism process | 1.16E-07 | 1.04E-05 |
| GOBP_ response to BMP | 1.26E-05 | 0.0006 |
| GOBP_ transmembrane receptor protein serine threonine kinase signaling pathway | 5.01E-06 | 0.0003 |
| GOMF_ hormone activity | 3.55E-06 | 0.0002 |

**Supplementary Table S3: The correlation of 22 types of immune cells was calculated**

|  | B cells naive (r value) | p value |
| --- | --- | --- |
| B cells memory | -0.26 | 0.002 |
| T cells CD4 memory resting | 0.3 | 0.0003 |
| T cells CD4 memory activated | 0.17 | 0.042 |
| Monocytes | -0.23 | 0.005 |
| Macrophages M1 | 0.18 | 0.035 |

|  | B cells memory (r value) | p value |
| --- | --- | --- |
| T cells CD4 naive | 0.46 | 7.68E-09 |
| T cells CD4 memory resting | -0.29 | 0.0003 |
| T cells regulatory (Tregs) | 0.17 | 0.0440 |
| T cells gamma delta | 0.22 | 0.0086 |
| NK cells activated | -0.19 | 0.0197 |
| Monocytes | 0.21 | 0.0113 |
| Macrophages M1 | -0.19 | 0.0229 |
| Macrophages M2 | -0.34 | 2.19E-05 |
| Dendritic cells activated | -0.19 | 0.0235 |
| Mast cells resting | -0.3 | 0.0002 |
| Neutrophils | 0.3 | 0.0003 |

|  | Plasma cells (r value) | p value |
| --- | --- | --- |
| NK cells activated | 0.27 | 0.0010 |
| Macrophages M2 | -0.12 | 0.0036 |
| Neutrophils | -0.13 | 0.0484 |

|  | T cells CD8 (r value) | p value |
| --- | --- | --- |
| T cells CD4 naive | -0.18 | 0.0293 |
| Monocytes | -0.21 | 0.0115 |
| Macrophages M1 | 0.17 | 0.0357 |

|  | T cells CD4 naive (r value) | p value |
| --- | --- | --- |
| T cells CD4 memory resting | -0.32 | 7.98E-05 |
| T cells gamma delta | 0.26 | 0.0018 |
| NK cells activated | -0.24 | 0.0044 |
| Monocytes | 0.27 | 0.0010 |
| Macrophages M0 | 0.19 | 0.0242 |
| Macrophages M1 | -0.23 | 0.0045 |
| Macrophages M2 | -0.37 | 4.28E-06 |
| Mast cells resting | -0.4 | 5.44E-07 |
| Mast cells activated | 0.18 | 0.0309 |
| Neutrophils | 0.51 | 8.13E-11 |

|  | T cells CD4 memory resting (r value) | p value |
| --- | --- | --- |
| T cells regulatory (Tregs) | -0.22 | 0.0074 |
| T cells gamma delta | -0.19 | 0.0252 |
| NK cells resting | -0.26 | 0.0015 |
| Monocytes | -0.42 | 1.25E-07 |
| Macrophages M0 | -0.39 | 1.55E-06 |
| Macrophages M1 | 0.27 | 0.0008 |
| Macrophages M2 | 0.44 | 2.21E-08 |
| Dendritic cells resting | 0.23 | 0.0046 |
| Mast cells resting | 0.3 | 0.0003 |

|  | T cells CD4 memory activated (r value) | p value |
| --- | --- | --- |
| NK cells resting | 0.21 | 0.0106 |
| Monocytes | -0.23 | 0.0054 |
| Macrophages M0 | 0.16 | 0.0478 |
| Macrophages M1 | 0.18 | 0.0267 |
| Mast cells activated | 0.17 | 0.0418 |

|  | T cells follicular helper (r value) | p value |
| --- | --- | --- |
| T cells gamma delta | 0.25 | 0.0027 |
| NK cells resting | -0.18 | 0.0331 |
| Monocytes | -0.18 | 0.0283 |
| Macrophages M1 | 0.19 | 0.0242 |
| Neutrophils | -0.17 | 0.0449 |

|  | T cells regulatory Tregs (r value) | p value |
| --- | --- | --- |
| NK cells activated | -0.18 | 0.0284 |
| Macrophages M0 | 0.19 | 0.0216 |
| Macrophages M2 | -0.26 | 0.0014 |
| Dendritic cells activated | -0.19 | 0.0200 |
| Neutrophils | -0.17 | 0.0426 |

|  | T cells gamma delta (r value) | p value |
| --- | --- | --- |
| NK cells resting | -0.2 | 0.0185 |
| NK cells activated | -0.21 | 0.0096 |
| Monocytes | 0.18 | 0.0294 |
| Macrophages M2 | -0.21 | 0.0094 |
| Mast cells resting | -0.2 | 0.0135 |
| Neutrophils | 0.18 | 0.0290 |

|  | NK cells resting (r value) | p value |
| --- | --- | --- |
| NK cells activated | -0.17 | 0.0372 |
| Monocytes | -0.16 | 0.0477 |
|  | NK cells activated (r value) | p value |
| Monocytes | -0.42 | 1.47E-07 |
| Macrophages M0 | -0.32 | 0.0001 |
| Macrophages M2 | 0.32 | 8.67E-05 |
| Dendritic cells activated | 0.17 | 0.0404 |
| Mast cells resting | 0.59 | 6.98E-15 |
| Neutrophils | -0.42 | 2.09E-07 |

|  | Monocytes (r value) | p value |
| --- | --- | --- |
| Macrophages M0 | 0.34 | 3.52E-05 |
| Macrophages M1 | -0.4 | 5.32E-07 |
| Macrophages M2 | -0.65 | 1.90E-18 |
| Dendritic cells resting | -0.34 | 3.72E-05 |
| Mast cells resting | -0.56 | 3.52E-13 |
| Neutrophils | 0.36 | 1.11E-05 |

|  | Macrophages M0 (r value) | p value |
| --- | --- | --- |
| Macrophages M2 | -0.59 | 8.70E-15 |
| Dendritic cells resting | -0.22 | 0.0075 |
| Mast cells activated | 0.25 | 0.0027 |
| Neutrophils | 0.28 | 0.0007 |

|  | Macrophages M1 (r value) | p value |
| --- | --- | --- |
| Macrophages M2 | 0.2 | 0.0178 |
| Dendritic cells resting | 0.22 | 0.0089 |
| Dendritic cells activated | -0.4 | 4.61E-07 |
| Mast cells resting | 0.27 | 0.0008 |
| Neutrophils | -0.3 | 0.0002 |

|  | Macrophages M2 (r value) | p value |
| --- | --- | --- |
| Dendritic cells resting | 0.25 | 0.0024 |
| Dendritic cells activated | 0.23 | 0.0051 |
| Mast cells resting | 0.44 | 2.36E-08 |
| Neutrophils | -0.47 | 1.84E-09 |

|  | Dendritic cells resting (r value) | p value |
| --- | --- | --- |
| Mast cells resting | 0.19 | 0.0231 |
| Neutrophils | -0.19 | 0.0210 |

|  | Mast cells resting (r value) | p value |
| --- | --- | --- |
| Neutrophils | -0.59 | 4.39E-15 |
