# Supplementary material for: Association of the LEP gene with immune infiltration as a diagnostic biomarker in preeclampsia
Source: Front Mol Biosci. 2023 Aug 10;10:1209144. doi: 10.3389/fmolb.2023.1209144 (PMC10448764; doi:10.3389/fmolb.2023.1209144)
Supplement: Supplementary file 2 [file DataSheet1.docx]

<https://www.jianguoyun.com/p/DYdGLuUQ_5-oChjbhLAEIAA>
